# Biological degradation of catechol in wastewater using the sequencing continuous-inflow reactor (SCR)

**DOI:** 10.1186/2052-336X-11-3

**Published:** 2013-05-24

**Authors:** Ali Ahmad Aghapour, Gholamreza Moussavi, Kamyar Yaghmaeian

**Affiliations:** 1 Department of Environmental Health Engineering, Faculty of Medical Sciences, Tarbiat Modares University, Tehran, Iran; 2 Department of Environmental Health Engineering, School of Public Health, Tehran University of Medical Sciences, Tehran, Iran

**Keywords:** Catechol, Sequencing continuous-inflow reactor, SCR, Biodegradation, Mineralization, HRT

## Abstract

Catechol is used in many industries. It can be removed from wastewater by various methods but biological processes are the most superior and commonly used technology. The SCR is a modified form of SBR used to degrade catechol. The objective of this study was to investigate the performance of SCR for biodegradation and mineralization of catechol under various inlet concentrations (630–1500 mg/L) and hydraulic retention times (HRT) (18–9 h). This study used a bench scale SCR setup to test catechol degradation. The acclimation time of biomass for catechol at degradation at 630 mg/L was 41 d. The SCR operating cycle time was 6 h and the consecutive times taken for aerating, settling and decanting were 4, 1.5 and 0.5 h, respectively. This study investigated the effects of inlet catechol concentration (630–1560 mg/L) and HRT (18–9 h). The average catechol removal efficiencies in steady-state conditions of 630, 930, 12954 and 1559 mg/L of catechol were 98.5%, 98.5%, 98.2% and 96.9% in terms catechol and 97.8%, 97.7%, 96.4% and 94.3% for COD, respectively. SCR with acclimated biomasses could effectively remove the catechol and the corresponding COD from wastewater with concentrations of up to 1560, at the loading rate of 5.38 kg COD/m^3^.d and at a HRT of up to 13 h. The HRT was determined as an important variable affecting catechol removal from wastewater. Reducing the HRT to below 13 h led to reduced removal of catechol and COD.

## Introduction

Catechol, (a derivative of benzene and a phenolic compound) it has many applications in industry for example as a photographic developer, lubricating oil, polymerization inhibitor and in pharmaceuticals [1,2]. Catechol has a strong aroma; it is also a toxic and persistent water pollutant in the environment [1]. The International Agency for Research on Cancer (IARC) has classified catechol in terms of a carcinogenic risk to humans (Group 2 B) [1]. Phenolic compounds such as catechol have been listed as priority-pollutants by EPA, USA [2,3]. Catechol is fatally toxic to fish at concentrations of 5–25 mg/L and it inhibits biological growth in microorganisms [4,5]. It has been detected in wastewater from coal conversion processes, crude wood tar, and drainage water from bituminous shale and in the outflow from coal-tar chemical production. Its concentration varies from a few mg/L to 2000 mg/L in wastewater from coal carbonization and gasification. At low temperature wastewater from coal carbonization, its concentration may be as high as 5300 mg/L [2,6,7]. To protect against the hazards of catechol to public health and the environment, catechol bearing aqueous waste must be treated with an efficient, cost effective and environmentally benign technique before being discharged [8,9].

A review of the related literature indicates that most investigations reporting on catechol removal from wastewater have focused on physical, chemical and biological processes. Physical processes such as adsorption transfer of pollutants from a liquid to a solid phase involve further treatment of the by-products. Chemical processes such as photocatalysis, Fenton, photo-Fenton and Ozonation processes are very efficient but expensive methods available for the mineralization of catechol. However, these chemical processes are often incomplete and create by-products that contribute to serious health risks and environmental hazards [1,10-12]. Biological processes have several technical advantages for treating biodegradable waste including greater cost-effectiveness, simplicity of implementation and operation and reliability, they are also environmentally benign with a high capacity for degradation [11]. In these terms biological processes are superior to other technologies such as physical and chemical processes used to treat biodegradable contamination.

Some researchers have studied the degradation of catechol in anaerobic bioreactors including up flow fixed film–fixed bed bioreactors [13] and UASB [2,6] acclimated to this compound. In the up flow fixed film–fixed bed reactor, at the loading rate of 4.79 kg catechol/m^3^.d, percentages for maximum degradation and COD removal of catechol were 93.11% and 90.81%, respectively [13]. The biodegradation of catechol through co-metabolism with glucose in an upflow anaerobic sludge blanket (UASB) reactor achieved 95% COD removal efficiency with 500–1000 mg/L catechol concentration in the feed and a glucose concentration of 1500 mg/L as a cosubstrate [2]. Due to the greater inhibitory effect of phenolic compounds on anaerobic microbial metabolism, aerobic biological methods are conventionally preferred to anaerobic biological methods for the treatment of wastewater [10].

Aerobic microorganisms are able to degrade organic toxic compounds more efficiently because they have higher growth rates than anaerobes and usually covert organic compounds to inorganic compounds (CO2, H2O) [10]. Many groups of aerobic bacteria are capable of using aromatic compounds as sole carbon and energy sources. There have been several reports on the biodegradation of catechol by some microbial strains under aerobic conditions such as *Pseudomonas putida*[4], *Aspergillus awamori*[14] and *Candida parapsilopsis*[5]. The bacterial strain *Pseudomonas putida* in a basal salt medium (BSM) at 29.9°C and pH 7 demonstrated the complete degradation of 500 mg/L of catecholin 94 h [4]. *Candida parapsilopsis*, in a standard medium, demonstrated the complete degradation of catechol at the concentration of 910 mg/L within 48 h [5].

However, research has shown that mixed microbial communities are more efficient than pure cultures in terms of their inhibitory effect of organic compounds in wastewater because of the various different organisms present, all of which are necessary for the degradation process [15]. Additionally, the aerobic metabolism is usually the most rapid biodegradation process for biodegradable pollutants [10,16].

Among those bioreactors invented to treat biological wastewater, SBR has some major advantages such as flexibility, robustness, single basin operation, better control of shock loads, simplicity of operation, relatively low cost and no sludge loss in the reaction period and hence no need to return the activated sludge [3,17,18]. SBR is therefore the most commonly applied type of biological process used to treat industrial wastewater [3,19,20]. When SBR is used to degrade toxic and inhibitory compounds, batch feeding of the substrate is a main defect for SBR because it affects the biodegradation rate of the substrate [11,21]. Nonetheless, due to the batch feeding mode, it requires multiple reactors to treat a continuous inflow [21].

Research by Moussavi et al. (2010) has recently begun to investigate efforts to modify SBR to overcome its defects [21]. Therefore, the mode of feeding the SBR modified from batch setting to continuous, showed a modification procedure to recuperate its performance in degrading toxic and inhibitory compounds through an adjustment of the loading peak and shock, as well as in allowing continuous treatment of wastewater in a single-basin bioreactor [11,21]. Since the SCR has only recently been introduced and demonstrates highly effective performance in the degradation of phenol and formaldehyde [21] and a mixture of formaldehyde and ammonia [11], it is considered worthwhile to examine the capability of this process of degradation of other substances with resistance to organic material including catechol that is frequently found in waste streams.

So, the objective of this study was to investigate the performance of SCR for biodegradation and mineralization under various inlet catechol concentrations (630–1500 mg/L) and hydraulic retention times (HRT) (18–9 h). The performance of SCR was evaluated in terms of degradation and chemical oxygen demand (COD) in the process of removing catechol from contaminated water.

## Materials and methods

### Wastewater preparation

Feed synthetic wastewater was prepared daily by dissolving catechol (Merck >99%) in tap water and adding aliquots of stock nutrient solution. The stock nutrient solution was prepared by dissolving 5 g K2HPO_4_, 15 gr KH_2_PO_4_, 120 g NH_4_Cl, 12 g (NH_4_)_2_HPO_4_, 10 g CaCO_3_ and 10 g NaHCO_3_ in one liter of tap water. Catechol was used as sole sources of carbon and energy and NH_4_Cl, K_2_HPO_4_, KH_2_PO_4_ and (NH_4_)_2_HPO_4_ were used for sources of nitrogen and phosphorous in the biomass in the SCR. The COD: N: P ratio in the feed wastewater was kept constant at 100:5:1 for all tests.

### Experiment setup

**Figure 1:**
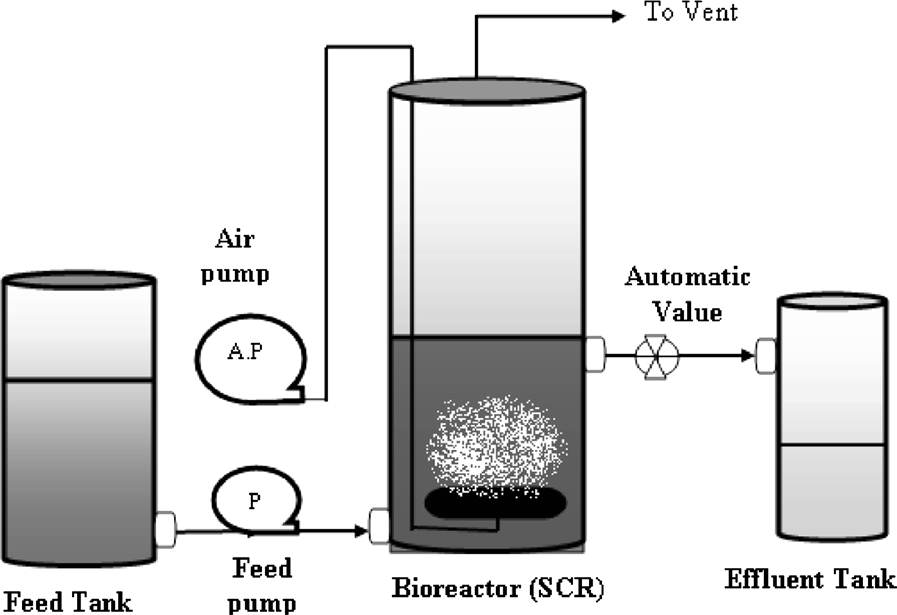
Flow diagram of the SCR experimental setup.

The bench scale SCR experimental setup used for these tests (Figure 1) was consisted of a glass cylindrical bioreactor with an inner diameter of 20 cm and total height of 36 cm, a wastewater feeding pump, an air injection pump, a supernatant decant system, a timing switch and other necessary accessories. A decant automatic time-controlled valve was located at the height of 10 cm from the bottom of the column giving a constant 3.14-L volume of mixed liquor remaining in the reactor at the end of the decant phase in each operating cycle.

### Biomass acclimation and reactor start-up and operation

At the beginning of the reactor start-up, the SCR was inoculated with 1.5 L of an acclimatized phenol degrading biomass [3] and 1.5 L of an acclimatized furfural degrading biomass [22], making the suspended solid concentration of 5000 mg/L in the SCR and operational in a continuous current condition. As the seed biomasses were already acclimated to phenol and furfural, the acclimation of biomass to catechol began with 600 mg/L furfural, then the concentration of furfural in the feed wastewater was gradually reduced and catechol was correspondingly increased to an approximate level of 600 mg/L. The starting time was considered to be complete when 95% removal of 630 mg/L catechol was obtained in the SCR.

After successful start up had been achieved, the effect of inlet catechol concentration (630 mg/L) and HRT (18 and 9 h) at each run, the concentration of biomass (measured at the end of decant phase) was 10 ± 1 g/L and kept constant over the course of the experiment by discharging the mixed liquor. The retention time for sludge in the bioreactor was calculated to be 21 d and that was kept constant throughout the tests in the study. The pH level of the feed wastewater was maintained at the value of around 7.2 ± 0.4 (the optimum value for bacterial growth) by adding CaCO_3_ and NaHCO_3_. The reactor was operated at room temperature (20 ± 2°C) over the course of tests.

The bioreactor was run for 168 days and operated in cycles. Each operating cycle time was 6 h and consisted of aerating, settling and decanting. The inlet wastewater was continuously fed in all cycle phases and the treated stream was decanted simultaneously throughout the decant cycle. Consecutive times for aerating, settling and decanting phases were 4, 1.5 and 0.5 h, respectively. The period of settling and decanting was kept constant over the course of study. The bioreactor was operated in two phases, the first phase of operation evaluated the effect of inlet concentrations at a constant HRT of 18 h and the second phase of the operation evaluated the effect of HRT (18, 13 and 9 h) at the constant catechol concentration of 1560 mg/L. These test phases and the SCR operation schedule are shown in Table 1.

**Table 1:** Experimental phases and SCR operation schedule

**Phases**	**Run**	**Day**	**Catechol inlet (mg/L)**	**COD inlet (mg/L)**	**Flow rate (L/d)**	**HRT (h)**	**OLR**
							**kg COD/m**^**3**^**.d**
Reactor start-up	0	0-35	630	1183	6.28	18	1.52
Effect of catechol concentration	A	35-47	630	1183	6.28	18	1.52
	B	48-80	930	1750	6.28	18	2.21
	C	81-90	1294	2433	6.28	18	3.17
	D	91-124	1560	2933	6.28	18	3.82
Effect of HRT	D	91-124	1560	2933	6.28	18	3.82
	E	125-158	1560	2933	10.67	13	5.38
	F	159-166	1560	2933	25.12	9	7.82

### Analysis

The inlet and outlet of the reactor were sampled daily and analyzed for catechol, COD, pH and TSS parameters. In order to measure catechol and COD, samples were filtered to remove particles before analysis using a Whatman filter with a 0.45 μm pore size. Concentrations of catechol were measured using the 5530D method of Standard Methods [23]. The mineralization rate of catechol was determined by COD measurements [23]. Concentrations of total suspended solids (TSS) in the mixed liquor were measured according to standard methods at regular intervals. All parameters were measured according to standard methods [23]. The pH level was measured using an electrode.

## Results and discussion

### Bioreactor start-up

**Figure 2:**
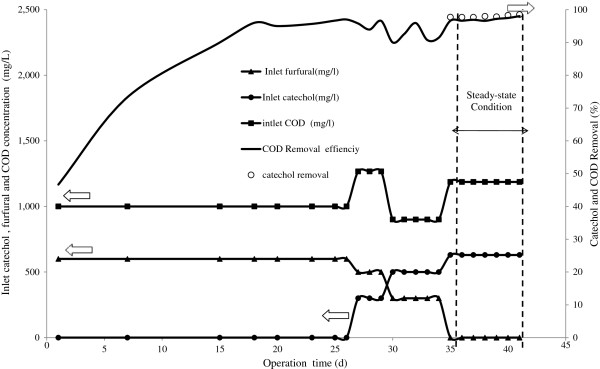
Catechol and COD removal percentages during the SCR start-up phase.

The SCR was started up by feeding it with synthetic wastewater (containing 630 mg/L furfural) for the cycle period of 6-h. Figure 2 shows the performance of the SCR during the start up period. As seen in Figure 2, after 15 days the SCR reached steady-state start-up and catechol and COD removal efficiencies were 95.9% and 95.7%, respectively. Then the operation of the reactor was continued until steady-state performance was reached. A steady state was assumed when both the catechol and COD removal efficiency remained constant (with changes of less than 3%) over a week’s period of operation [8,24]. This reveals that the biomass SCR has acclimated to catechol and successful start up has been reached. During the acclimatization process certain enzymes are induced in the microorganisms that participate in the biodegradation reaction. This is more significant when dealing with high concentrations of toxic compounds such as catechol [4]. Enzymes in the degradation of catechol aromatic substrate are synthesized in appreciable amounts only when the substrate or similar structural compounds are present [15]. In an aerobic condition, the biomass acclimation period may vary from several hours to several days [25]. In this study, the acclimation time of biomass for catechol was 41 d. The MLSS concentration was fixed in 10 ± 1 g/L after day 41 of the operation.

### Effect of inlet catechol concentration

**Figure 3:**
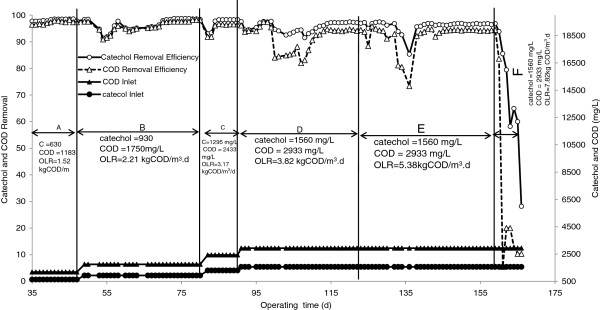
Profile of the catechol and COD removal efficiencies at various inlet concentrations.

The SCR operation was the study of the inlet catechol concentration effect ranging from 630 to 1560 mg/L. Figure 3 depicts the degradation of catechol and data relating to the timing of COD removal. As observed in Figure 3, when switching the inlet catechol concentration to a higher level (zone A to D), the performance of SCR initially decreased but it recovered and reached steady-state after a few days of changing the concentration.

**Figure 4:**
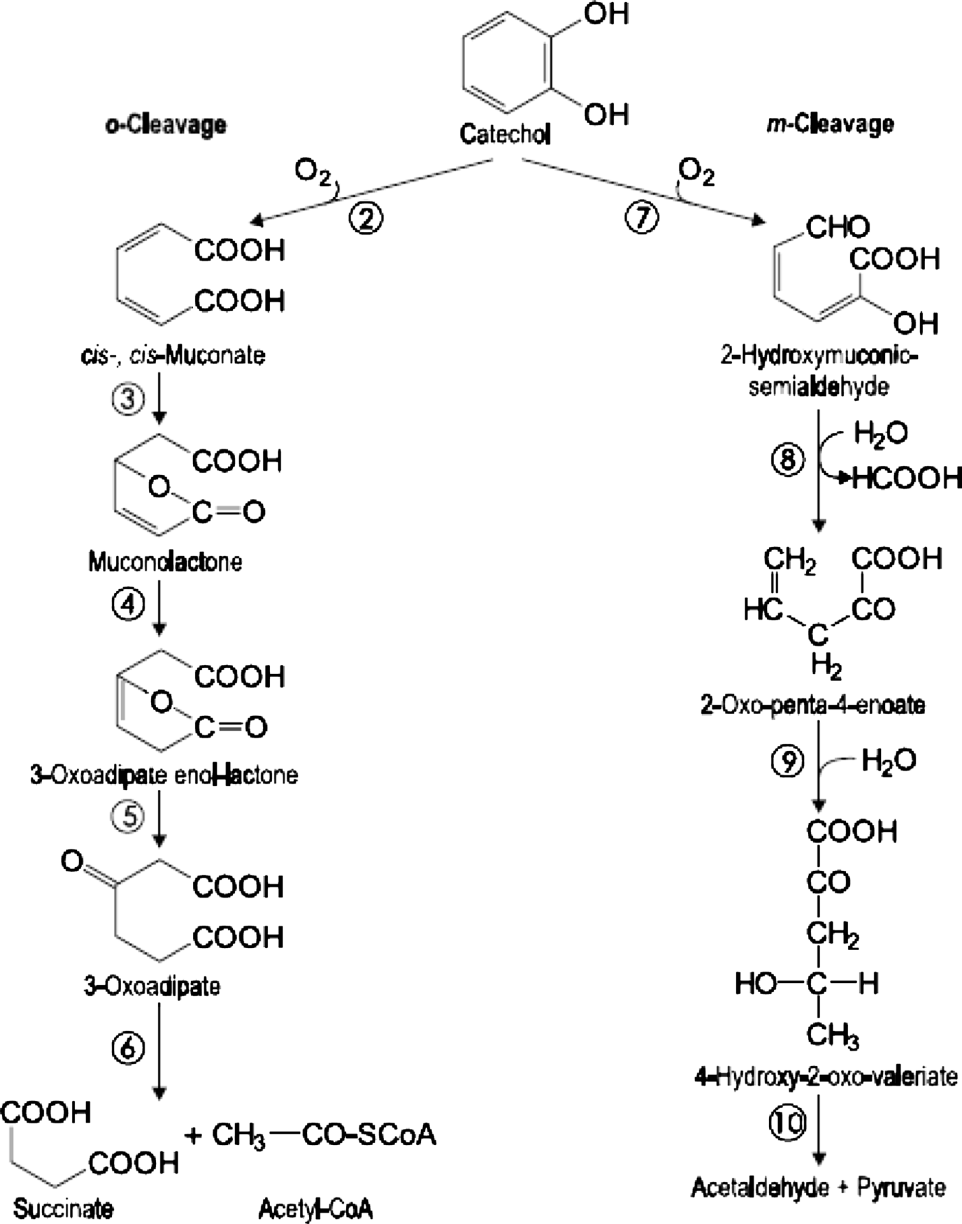
**The ortho and meta cleavage pathways in aerobic degradation of catechol [**[15]**].**

The sudden decrease in efficiency of COD and catechol removal with an increase of catechol concentration may be due to inhibition of catechol on the process of microbial metabolism [12,26]. The higher the inlet catechol concentration, the greater is the inhibitory effect and thus the greater reduction rate of degradation resulting in a longer recovery period. For example, percentages of catechol degradation were reduced to 91%, 93% and 91% when the inlet concentration was adjusted from 630 to 960 mg/L then to 1295, and finally to 1560 mg/L, respectively. Also, the recovery period (time to reach the steady state) for inlet catechol concentrations of 630, 960, 1295 and 1560 mg/L were 20, 10 and 30 days. Furthermore, as seen in Figure 3, the degradation of catechol was higher than its COD removal for higher inlet concentrations. The lower COD removal than that of catechol may be related to the degradation pathway of catechol. Under aerobic conditions, the ortho and meta cleavage pathways are two typical pathways for metabolizing the catechol compound [10]. The ortho and meta cleavage pathways are shown in Figure 4[15].

**Figure 5:**
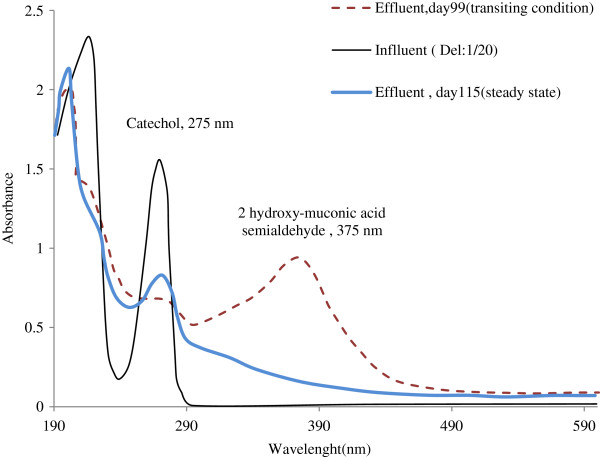
The average catechol and COD removal efficiency at steady-state condition as a function of inlet concentration.

In an aerobic degradation process, most of the microorganisms typically prefer the ortho pathway [27]. The ortho-cleavage pathway (intradiol fission) is more effective than the meta-cleavage pathway in terms of converting carbon to cell mass [28]. While meta-cleavage (extradiol fission) is generally used when ortho-cleavage cannot be facilitated [28]. As showed in Figure 4, when the initial ring fission is accomplished by the ortho cleaving pathway, the product of ring fission is a cis-cis-muconic acid, when this is accomplished by the meta cleaving pathway, the product is 2 hydroxy-muconic acid semialdehyde [29]. 2 hydroxy-muconic acid semialdehyde is yellow and has its maximum absorption wavelength at 375 nm. So, the creation of yellow in the reactor and an increase in the absorption wavelength at 375 nm in the effluent (Figure 5) reveals that catechol was degraded in the SCR via the meta cleaving pathway to 2 hydroxy-muconic acid semialdehyde under the selected condition [30-36].

However in the steady-state condition (days 115–122 of the operation), the effluent was colorless and the absorption wavelengths at 375 nm were very low (Figure 5). It can therefore be inferred that at the steady-state condition, catechol was degraded via the ortho cleaving pathway, while transiting the concentration of catechol conditions, degradation was probably accomplished via both ortho and meta cleaving pathways. Accordingly, the higher effluent concentrations of COD than of catechol at higher inlet concentrations can be attributed to the formation of organic intermediate compounds, as shown in Figure 4[15].

**Figure 6:**
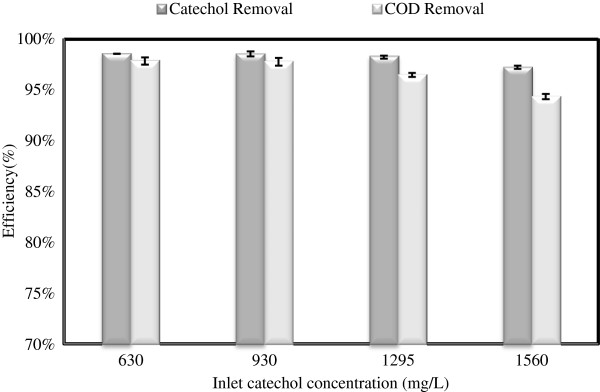
The average catechol and COD removal efficiency at steady-state condition as a function of the inlet concentration.

To understand the influence of inlet catechol concentration of the performance of SCR, the average steady state of catechol and its COD removals were obtained and are shown in Figure 6. As observed in Figure 6, the average catechol removal efficiency on steady-state condition at catechol levels of630, 930, 12954 and 1559 mg/L were 98.5%, 98.5%, 98.2% and 96.9% in terms of catechol and 97.8%, 97.7%, 96.4% and 94.3% as COD, respectively. As demonstrated, the steady state degradation and COD removal of catechol were negligible at different inlet concentrations (<3%). These findings indicate that SCR is capable of degrading high concentration catechol and that the level of inlet catechol concentration has no considerable detrimental effect on the performance of SCR.

**Figure 7:**
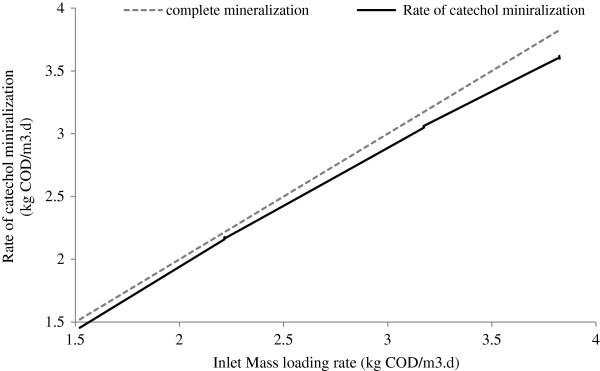
Mineralization of catechol in phase I at various mass organic loading.

Figure 7 shows the rate of catechol mineralization at various inlet mass loading rates. The rate of catechol mineralization increased correspondingly with an increased loading rate of the inlet. Also, the lines of mineralization indicate a greater deviation from the line of complete mineralization with an increased rate of inlet loading.

Nonetheless, the SCR attained mineralization of 3.60 kg COD/m^3^.d at the maximum applied inlet load of 3.82 kg COD/m^3^.d. This indicates that the capacity of SCR for mineralization of catechol is greater than the level obtained. No work could be found in the available literature on the aerobic biodegradation of catechol in SCR. There are currently only a few studies on the anaerobic biological degradation of catechol by UASB and upflow fixed film–fixed bed reactors. Latkar et al., (2003) reported that the maximum substrate removal of 93.11% and COD removal of 90.81% in upflow fixed film–fixed bed reactors at the catechol concentration of 750 mg/L, the loading rate of 4.79 kg catechol/m^3^.d and the HRT of 6 h [13]. Research by Subramanyam and Mishra [2] investigated the biodegradation of catechol through co-metabolism with glucose by UASB reactor and demonstrated a rate of more than 95% COD removal efficiency when the reactor feed contained catechol in concentrations ranging from 500 to 1000 mg/L (OLR=2.86-5.7 kg COD m^3^/d) with a constant glucose concentration of 1500 mg/L and a constant HRT of 8 h. Their another study [6] also demonstrated the performance of the UASB reactor for the co-degradation of catechol and resorcinol at a total concentration of 1000 mg/L (800 mg/L of catechol and 200 of mg/L resorcinol) with a fixed organic loading rate of 5.7 kg COD/m^3^d and with a HRT of 8 h and determined a removal efficiency of 92% as COD.

According to the literature, most investigations in to biodegradation of catechol have reported on tests under anaerobic conditions and/or through co-metabolic degradation processes, whereas biodegradation of catechol in SCR was accomplished by growth-linked biodegradation metabolism under aerobic conditions. In a growth-link biodegradation process, organic compounds such as catechol are used as sole sources of carbon and energy. This growth based process for biodegradation is the most rapid and complete method for the treatment of most organic pollutants such as catechol and the process results in the complete degradation of catechol compared with the co-metabolic degradation process [15]. The co-metabolic degradation process is the basis of bio transformations or bioconversions and the process converts a substance to a chemically modified form [15]. Furthermore, aerobic degradation processes have always been considered preferable to anaerobic degradation in terms of kinetics and capacity [37]. Therefore, the SCR as an aerobic process transformed catechol to inorganic and environmentally acceptable compounds, whereas in an anaerobic condition, like UASB, it may be converted to incomplete and smelly compounds and may require further treatment. So, from a comparison of the results of tests in this study with the results reported in available literature, it can be induced that SCR performed much better in terms of degradation and mineralization of catechol than other processes.

### Effect of hydraulic retention time

To investigate the effect of HRT on biodegradation and COD removal of catechol using SCR, the inlet flow rate was increased in steps from 6.28 to 25.12 L/d and then related to the HRTs of 18 to 13 and 9 h. The catechol concentration in the feed wastewater was kept at a constant 1560 mg/l. The time-course results for degradation and COD removal are shown in Figure 6 (zones D, E and F). As demonstrated in Figure 6, upon switching the SCR operation from HRT of 18 h to 13 h, (zone D to zone E corresponding to day 124 of the operation), catechol degradation and COD removal were at first slightly reduced to percentages of 83% and 73% respectively. As demonstrated in Figure 6 these sudden decreases in COD and catechol removal efficiencies at this zone were harsher than they had been in the previous zones. Similar to other switching conditions, a yellow color was observed in the bioreactor and the absorption wavelengths of the bioreactor outlet were increased at 375 nm. However, on continuing operation of the bioreactor there was a rapid improvement to its performance and steady-state performance was attained on day 159. A longer HRT duration to 9 h resulted in progressive reduction of degradation and COD removal of catechol. As can be seen in zone F of Figure 6, the rate of catechol degradation decreased to below 30% after 7 days upon HRT reduction. This suggests that the level of catechol loading on SCR was over the tolerable limit and thereby inhibited metabolism in the microbial degradation process. Accordingly, an HRT duration of 13 h, corresponding to an organic loading of 5.38 kg COD/m^3^, at which the COD removal 94% was selected as the optimum level for loading on the SCR. As mentioned earlier, Latkar (2003) reported that using upflow fixed film–fixed bed reactors could remove catechol and COD corresponding to 93.11% and 90.81% at the HRT of 6 h, concentrations of 750 mg/L and the loading of 4.79 kg catechol/m^3^/d. Subramanyam and Mishra [2] reported more than 95% COD removal efficiency through co-metabolism with glucose by UASB reactor at a HRT of 8 h, catechol concentration inlet range of 500–1000 mg/L, OLR=2.86-5.7 kg COD m^3^/d and with a constant glucose concentration of 1500 mg/L. Therefore, the findings of this study compared to those of the related literature indicated that SCR had greater biodegradation efficiency than that of UASB [2] and upflow fixed film–fixed bed reactors [13].

## Conclusions

This work investigated the performance of SCR for degradation and COD removal of catechol from wastewater. The conclusions drawn from this work are summarized as follows:

• The performance of SCR was not considerably affected by inlet catechol concentrations up to 1560 mg/L, with a loading rate of 3.82 kg COD/m^3^.d and at the HRT of 18 h.

• The HRT is an important variable affecting catechol removal from wastewater in the SCR, in that reducing the HRT to below 13 h led to reductions in levels of catechol and COD removal.

• The SCR with acclimated biomasses could efficiently eliminate over 96% and 94% of catechol and COD up to inlet loads of 5.38 kg COD/m^3^.d and at a HRT of up to 13 h.

## Competing interests

The authors declare that they have no competing interests.

## Authors’ contributions

All authors have made extensive contribution into the design, experiments and data analysis, manuscript preparation, review and finalization of this manuscript. All authors read and approved the final manuscript.
